# Incidental Laparoscopic Discovery of an Intraperitoneal Plastic Catheter 16 Years after an Unsafe Abortion: A Case Report from the Gynecologic, Obstetric, and Pediatric Hospital of Yaoundé (Cameroon)

**DOI:** 10.1155/2017/7130479

**Published:** 2017-10-09

**Authors:** Ngandji Andre, Ngo Um Meka Esther Juliette, Joel Fokom Domgue, Wandji Brigitte, Foumane Pascal

**Affiliations:** ^1^Department of Gynecology and Obstetrics, Faculty of Medicine and Biomedical Sciences, University of Yaoundé I, Yaoundé, Cameroon; ^2^Department of Gynecology and Obstetrics, The Yaoundé Gyneco-Obstetric and Pediatric Hospital, Yaoundé, Cameroon

## Abstract

In many developing countries like Cameroon, unsafe abortion is a major public health problem. It can be responsible for severe complications including damage to the digestive and/or urinary tract, sepsis, and uterine perforation. Uterine perforation could be caused by most of the instruments that are used to evacuate the uterus. We report a case of apparent uterine perforation and subsequent migration of the plastic or rubber catheter into the peritoneal cavity during an abortion procedure performed in a setting that may have been unsafe. The discovery was made during a diagnostic laparoscopy indicated for secondary infertility of tubal origin 16 years after the abortion procedure. This is a rare clinical finding which is of therapeutic and diagnostic importance. To the best of our knowledge, a single similar case has been reported so far in the literature.

## 1. Introduction

Unsafe abortion is defined by the World Health Organization as a procedure for terminating an unwanted pregnancy either by persons lacking the necessary skills or in an environment lacking minimal medical standards or both [1]. It is a major public health problem in many developing countries like Cameroon where it is responsible for severe complications including damage to digestive and/or urinary organs, sepsis, and uterine perforation. Uterine perforation could be caused by most of the instruments that are used to help evacuate the uterus. In 2014, a study from India has reported the usage of rubber catheters as instruments to empty the uterus [2]. We report a case of uterine perforation by a plastic catheter and secondary migration of the catheter into the peritoneal cavity. The intraperitoneal catheter was discovered incidentally during a diagnostic laparoscopic procedure indicated for secondary infertility of tubal origin, 16 years after the abortion procedure was performed.

## 2. Case History

A 33-year-old patient, G3P2011, consulted at the Yaoundé Gyneco-Obstetric and Pediatric Hospital in 2015 for difficulty to conceive for 5 years. Before coming to us, she did not report any prior consultation for this problem. In her past medical history, she had undergone a clandestine abortion of a 12-week pregnancy about 16 years before that was carried out in a primary health facility by a nurse. She could not recall the detailed procedure that was performed, but she said that the nurse inserted something into her vagina to empty the uterus. Then she took some medication for a couple of days after the procedure that was condoned by her parents. The post abortum period was normal with no hemorrhage nor infection.

Later on, she had two normal vaginal deliveries at term with no maternal or fetal complications, about 2 years and 9 years after the abortion. At some point, she was also diagnosed with a genital* Chlamydia trachomatis* infection that was treated with oral medication. Physical examination was unremarkable.

A transvaginal ultrasound revealed a nongravid uterus with 2 subserosal myomas both 9 mm in diameter and stigmata of chronic bilateral adnexitis. Then, a hysterosalpingogram found a right distal tubal obstruction while her partner's spermogram was normal. Based on these findings, our working diagnosis was a secondary infertility probably of tubal origin.

**Figure 1:**
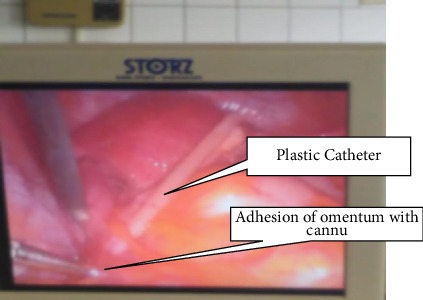
Laparoscopic image showing the foreign body buried in omental adhesion.

**Figure 2:**
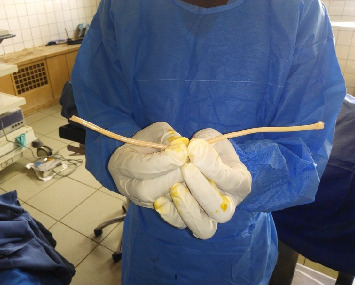
Foreign body after extraction.

The goal of our management was to repair tubal damage and enable her to get pregnant spontaneous. We carried out an exploratory laparoscopy and our findings were as follows: Fitz-Hugh-Curtis perihepatic adhesion; an intraperitoneal foreign body that was buried in the omentum (Figure 1). The foreign body looked like a plastic catheter, which was 30 cm long and 0.5 cm thick (Figure 2). The left fallopian tube was buried in a thick type C adhesion with the uterus and the omentum. The right fallopian tube was also buried in a thick C adhesion with the right ovary, the posterior uterine wall, and the omentum. Chromopertubation test was negative and the Mage's fallopian tube score was III-IV on both sides. We thus carried out a massive adhesiolysis which freed both tubes and did a bilateral neostomy. Given the very low likelihood of spontaneous pregnancy, she was referred for assisted reproduction.

## 3. Discussion

In developed settings, voluntary termination of pregnancy has been legalized and has led to the reduction of complications associated with this procedure, while illegal abortion is still a major problem in developing countries where it often gives rise to fatal complications.

In Cameroon, voluntary termination of pregnancy is prohibited unless there is a medical condition threatening maternal life, in case of incest or rape [3]. Illegal abortions are usually carried out clandestinely, by untrained personnel often with inadequate material, and therefore can lead to the numerous aforementioned complications.

Uterine perforation due to an induced abortion has been estimated at 0.8 per 1000 cases [4]. The culprit instruments could include, but are not limited to, safety pins, sticks, plant roots, and plastic cervical dilators [5]. In our case, the perforation was done during an illegal abortion using a plastic catheter.

A similar case with secondary migration in the peritoneal cavity has been reported in India [6]. To the best of our knowledge, no previous case has been reported in Cameroon and Africa.

After uterine perforation, if the instrument is left inside the uterus it usually migrates cranially into the peritoneal cavity. This probably happens because there is an initial partial perforation in a relatively avascular zone and if proper antibioprophylaxis is done, infection is thwarted. The uterine involution and retraction which occur later will complete the perforation process and favour migration into the abdomen. The slow pace of the process explains the paucity of digestive symptoms.

Clinically, the time to discovery is very variable, from a few days to several years depending on the interventions [6]. The symptoms when present could range from sensation of a mass, abdominal pain, nausea, vomiting due to subocclusion, hematochezia, diarrhea, and signs like fever and weight loss.

Other reported outcomes include presence of intra-abdominal abscess, peritonitis, foul smelling vaginal discharge, chronic fistulae, and granulomas presenting as digestive tumors and digestive hemorrhage due to vascular lesions by the migrating object [7].

Our patient was asymptomatic; the foreign body was discovered incidentally during a laparoscopy for secondary infertility of tubal origin. Removal of such objects is recommended because of the risk of injury to nearby organs or severe inflammatory reaction [6]. Usually it is found floating in the pouch of Douglas or buried in thick adhesion with the sigmoid colon and omentum like in our case. Laparoscopy is the best curative approach. This was carried out in our patient, though initially planned for a different indication. In case of difficulty or failure, laparoscopy could eventually be converted into laparotomy.

## 4. Conclusion

Uterine perforation is a common complication of unsafe abortion. It can be caused by a plastic catheter like in this case. If the catheter is left in place, it is taken up and migrates into the peritoneal cavity. It is therefore necessary for providers to be well trained about the abortion procedure and to patients to be well informed of the details of such procedures and all incidental happenings.
