# Corrigendum to “Incidental Laparoscopic Discovery of an Intraperitoneal Plastic Catheter 16 Years after an Unsafe Abortion: A Case Report from the Gynecologic, Obstetric, and Pediatric Hospital of Yaoundé (Cameroon)”

**DOI:** 10.1155/2020/1875314

**Published:** 2020-10-05

**Authors:** Ngandji Andre, Ngo Um Meka Esther Juliette, Joel Fokom Domgue, Wandji Brigitte, Foumane Pascal

**Affiliations:** ^1^Department of Gynecology and Obstetrics, Faculty of Medicine and Biomedical Sciences, University of Yaoundé I, Yaoundé, Cameroon; ^2^Department of Gynecology and Obstetrics, The Yaoundé Gyneco-Obstetric and Pediatric Hospital, Yaoundé, Cameroon

In the article titled “Incidental Laparoscopic Discovery of an Intraperitoneal Plastic Catheter 16 Years after an Unsafe Abortion: A Case Report from the Gynecologic, Obstetric, and Pediatric Hospital of Yaoundé (Cameroon)” [1], there was an error in the author list, where the author name “Fokom Joel” should have read “Joel Fokom Domgue”. This is corrected as shown above.
